# Activity rhythm and action range of workers of the invasive hornet predator of honeybees *Vespa velutina*, measured by radio frequency identification tags

**DOI:** 10.1002/ece3.4182

**Published:** 2018-07-08

**Authors:** Juliette Poidatz, Karine Monceau, Olivier Bonnard, Denis Thiéry

**Affiliations:** ^1^ UMR 1065 Santé et Agroécologie du Vignoble INRA Villenave d'Ornon France; ^2^ UMR CNRS 7372 Centre d'Etudes Biologiques de Chizé Université de la Rochelle Villiers‐en‐bois France

**Keywords:** Asian yellow‐legged hornet, central place foraging, early detection, invasive species, radio frequency identification, radio tracking, Vespidae

## Abstract

In social insects, the activity rhythm of foragers and their action range determinate the activity of the colony. In vespids, which are mostly predators, the foraging range of workers determines their maximum predation pressure round the nest. One of these species, *Vespa velutina*, a recently invasive species introduced into Europe, exerts a strong predation on honeybees at the hive. Therefore, the definition of its activity rhythm and spatial range of predation is of primary importance. Using radio frequency identification tags (RFID), two experiments were carried out to (a) determine their return ability (called homing) in releasing 318 individuals at different distance from their colony and (b) monitor their foraging activity rhythm and the duration of their flights based on 71 individuals followed 24 hr/24 during 2 months. The homing ability of *V. velutina* was evaluated to be up to 5,000 m and was not affected by the cardinal orientation of release point. The lag time to return to the nest increased with the distance of release. Most of the flight activity took place between 07:00 a.m. and 08:00 p.m., hornets doing principally short flights of less than an hour. Foraging range was thus estimated ca. 1,000 m around the nest. This study of *V. velutina* assisted by RFID tags provides for the first time a baseline for its potential foraging distance that increase our knowledge of this species to (a) refine more accurately models for risk assessment and (b) define security perimeter for early detection of predation on invasion front.

## 1 INTRODUCTION

Central place foraging is largely represented in animals in both vertebrates and invertebrates (Bell, 1990). It implies that individuals optimally explore a foraging area and are able to return to their nest after foraging for resources, to store, and to share it with the members of the group family (Bell, 1990; Houston & McNamara, 1985; Orians & Pearson, 1979). Nesting site choice results from the trade‐off among the habitability of the location, its safety from predators and the distance to resources (Osborne et al., 1999, 2008; Pyke, Pulliam, & Charnov, 1977; Williams & Kremen, 2007). To limit foraging costs, individuals optimize different parameters linked to foraging such as the distance they travel (Bell, 1990; Pyke, 1984). For example, bumblebees are able to adjust their trap lines linking different flowers in few foraging bouts to reduce the duration of the nectar collection (Lihoreau et al., 2012). One limiting key parameter is, however, the maximal distance an individual is able to travel going back home, called homing ability (Van Nieuwstadt & Ruano Iraheta, 1996). Homing ability is an intrinsic parameter of a species, while its actual foraging range depends on the resource distributions, abundance, and quality (Bacon et al., 1965; Rikkets, 2001), the individual ability (Greenleaf, Williams, Winfree, & Kremen, 2007), the landscape context (Southwick & Buchmann, 1995; Steffan‐Dewenter & Kuhn, 2003), and climatic parameters. Additionally, homing ability is also closely related to orientation. Studies on homing abilities of diverse organisms (insects and birds for instance) allowed the discovery of compass systems and include the use of the sun, the stars or geomagnetic fields (Collett & Collett, 2002; Gould, 1986; Goulson & Stout, 2001; Wehner & Menzel, 1990).

Homing ability and orientation have been extensively studied in social insects, mostly in pollinators such as honeybees (Abrol & Kapil, 1994; He et al., 2012; Pahl, Zhu, Tautz, & Zhang, 2011; Van Nieuwstadt & Ruano Iraheta, 1996) and bumblebees (Goulson & Stout, 2001). For example, *Bombus terrestris* workers basically forage in a 1,000‐m range, but may travel up to 4,300 m from their colony to collect valuable resources (Goulson & Osborne, 2009; Goulson & Stout, 2001; Osborne et al., 1999; Wolf & Moritz, 2008). They are, however, able to find their way home up to ca. 10,000 m, what is twice their maximal foraging range (Goulson & Stout, 2001). It is interesting that homing behavior and orientation have been rarely investigated in vespid species probably because their impact on ecosystems is less important than the pollination service provided by bees in general (see Schöne, Harris, & Mahalski, 1993; Schöne, Tengö, Kühme, & Kühme, 1993 on diggerwasp, Stürzl, Zeil, Boeddeker, & Hemmi, 2016 on groundwaps, Ugolini, 1985, 1986, 1987 on *Polistes* sp. and *Vespa orientalis*).

The Asian yellow‐legged hornet, *Vespa velutina var nigrithorax* (Lepelletier 1835), was accidentally introduced into Europe in 2004 from eastern China (Arca et al., 2015; see Monceau, Bonnard, & Thiéry, 2014 for a review). Since its introduction, it has spread through Europe in Spain (López, González, & Goldarazena, 2011), Italy (Demichelis, Manimo, & Porporato, 2013), Portugal (Grosso‐Silva & Maia, 2012), and Germany (Witt 2015) and more recently in UK, Belgium (2016), and Switzerland (2017). *Vespa velutina* is a generalist predator of arthropods mostly known for its damages on honeybee hives (Abrol, 1994; Shah & Shah, 1991; Tan et al., 2007). This predation pressure can directly and indirectly, by reducing the beehive overwintering abilities, enhance the colony loss risks by decreasing their foraging activity (Monceau et al., 2014; 2018). Thus, *V. velutina* predation is an additional pressure that contributes to bee decline (Goulson, Nicholls, Botias & Rotheray, 2015).

The life cycle of *Vespa velutina* is annual. During spring, a single gyne (foundress) initiates a nest and lays her eggs. Once the first workers emerge, they quickly replace the queen for all activities except egg laying. The colony grows through the months and the need for proteins to feed the larvae increases too, resulting in an increase in the predation on honeybee hives during summer and fall (Monceau, Maher, Bonnard, & Thiéry, 2013). In mid‐September–early October, males and gynes emerge, leave the nest, and mate. Only gynes (mostly mated, Poidatz, Bressac & Thiéry, unpublished data) hibernate during the winter, while the rest of the colony (males, workers, and the old queen) dies (Monceau et al., 2014). The nests of *V. velutina* can be found from underground to the top of the trees. They are paper made, often water hose shaped or spherical, with one unique small entrance. The study presented here took place during the intensive period of predation, in summer and beginning of fall.

To date, the action range of *V. velutina* is still unknown, while it is of first importance for the monitoring and the potential management of invasive species (Holway & Suarez, 1999). This information could help finding colonies and give a scale for potential control methods application. However, only models concerning nest distribution are available (Bessa, Carvalho, Gomes, & Santarém, 2016; Franklin et al., 2017; Monceau & Thiéry, 2017; Robinet, Suppo, & Darrouzet, 2016; Villemant et al., 2011). Risk assessment that integrates the action range of *V. velutina* is still missing but is unavoidable to progress in the management of this alien predator.

In order to accurately record the rhythm of entries and exits from the nest of several *V. velutina* workers at the same time over a long period of time, a *V. velutina* colony, maintained in semi‐field conditions, was equipped with radio frequency identification (RFID) device. Multiple release of tagged hornet allowed (a) evaluating the homing ability of *Vespa velutina*, and the part of cardinal orientation of the release points and body condition in this behavior, (b) describing the activity of the hornets at the individual level within the colony. Two main experiments were thus realized in parallel: (a) the release of individually tagged workers at increasing distance from the nest to measure their homing ability and (b) the assessment of daily individual activity for the workers released at the vicinity of the nest.

## 2 MATERIAL AND METHODS

### 2.1 Nest installation

A 15‐cm‐large‐diameter *V. velutina* wild colony was collected in St Médard‐en‐Jalles (Aquitaine, France, GPS coordinates: 44°53′35.8″N 0°44′51.4″W) on the 28 April 2016. After a 24‐hr cooling period at 4°C, the nest was carefully fixed with iron strings inside a cage (Supporting Information Appendix S1), made of mahogany stainless steel grid and Plexiglas (see Couto, Monceau, Bonnard, Thiéry, & Sandoz, 2014; Monceau, Bonnard, & Thiéry, 2013). The cage was then transported inside a 2 m × 1.5 m × 2 m stainless steel grid cabin with a corrugated plastic roof in the INRA de Bordeaux site (La Grande Ferrade, Aquitaine, France, 44°47′30.4″N 0°34′36.9″W). The nest was first installed with no possible outlet from the cage, with food, water, and nest construction material (wood, leaves, bark) provided *Ad libitum*, to prevent the colony from relocation. After a one‐week acclimatization period, a tunnel was installed to connect the cage to the outside (Supporting Information Appendix S1). The inner cage was covered with opaque cardboard sheets, to provide a single light source from the tunnel outlet, and help hornets to find the exit. At the same time, food previously provided inside the cage was removed. The colony could then grow freely for a week before the installation of the RFID system.

### 2.2 RFID system

As compared to other techniques such as radio tracking, the RFID technic has several main advantages: It is cheap, allows tagging individuals with a unique combination, and limits handling (Boiteau, Meloche, Vincent, & Leskey, 2009; Kissling, Pattemore, & Hagen, 2014). It was already used for homing studies especially in honeybees (Kissling et al., 2014).

Two RFID portals A and B (MAJA^®^ reader module 4.2, Mycrosensys) were placed in series on a wood support at the entrance of the tunnel on the outside (Supporting Information Appendix S2), thus recording AB sequence or BA sequence for on‐ or outgoing movements, respectively, that were recorded by a RFID HOST controller iID^®^ HOST MAJA (Mycrosensys) (see Henry et al., 2012; He et al., 2012 for details).

### 2.3 Hornet tagging

*Vespa velutina* workers were collected at their nest entry to be equipped with RFID micro‐TAG (mic3^®^‐TAG 16Kbit, iID‐2000‐G, 2.0 × 1.7 × 0.5 mm). The captured hornets were gently isolated in a falcon tube (50 ml) and then anesthetized by keeping the tube on ice for 15–20 min. Back to the laboratory, each hornet was immediately weighted (AS 220/C/2, Radwag 2011, precision ± 0.0001 g). The largest distance between the eyes was used as for a measurement of head width and obtained with an electronic caliper (precision ± 0.01 mm). Prior to fixation, the RFID micro‐tag was activated and then fixed on the dorsal side of the hornet thorax using temporary cement (TempoSIL2, Coltène). The tagged hornets were allowed to recover in groups of eight individuals on different meshed boxes (10 × 20 × 10 cm), with water and honey *ad libitum* during a maximum of 3 hr before their release, either next to the nest or farther for the homing experiment (see below). The monitoring of these tagged hornets was realized from the 8 August 2016 to the 11 November 2016.

### 2.4 Hornets release

**Figure 1:**
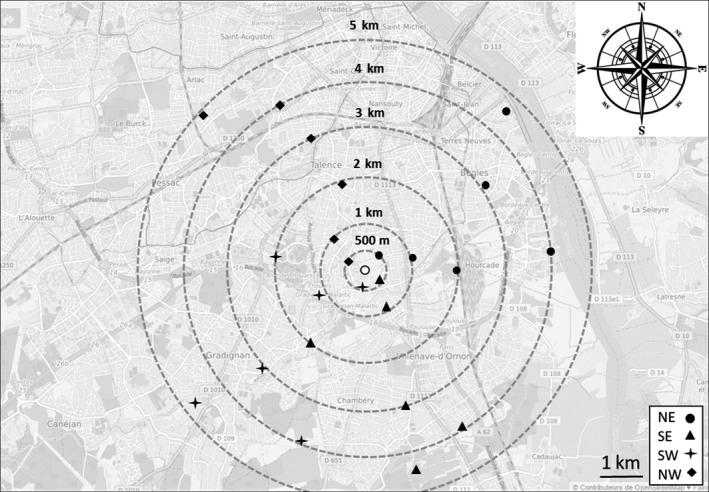
Map of the different release points of the hornet batches. The white dot in the center stands for the position of the nest. Rounds: NE = northeast; triangles: SE = southeast; crosses: SW = southwest; diamonds: NW = northwest. GPS coordinates are provided in Supporting Information Appendix S3. Background map Openstreetmap©

To test until which distance hornets are capable to return to their nest in field condition, called here homing ability, 318 workers of the same colony were released at different dates between 02:00 p.m. and 05:00 p.m. at four different places corresponding to the four cardinal points for each distance from their nest: being at 0, 500, 1,000, 2,000, 3,000, 4,000, and 5,000 m (Figure 1, Supporting Information Appendix S3). Traveling boxes with hornets were placed in an opaque plastic crate both to protect the hornets from heat and also to prevent them from getting any guiding visual information before release. To confirm first results, another release session was carried out for the distances of 3,000, 4,000, and 5,000 m. Care was taken to release the hornets in fair climatic conditions at each session: sunny days with average temperatures, no rain, no direct sunny exposure, and low wind.

To observe hornet activity, *that is,* the frequency of trips starting and ending at the colony, an additional batch of 41 workers was released near the nest on three consecutive days (8, 9, and 10 August 2016) between 02:00 p.m. and 05:00 p.m.

### 2.5 Data analysis

Statistical analysis was performed using R software (v.3.3.0., R Core Team 2016). First, the sessions 1 and 2 for the release distances of 3,000, 4,000, and 5,000 m were compared using either Fisher's exact test (3,000 and 5,000 m) or Pearson's *χ* ^2^ test (4,000 m). The standardized mass (i.e., the body condition) of the hornets was assessed with the scale mass index developed by Peig and Green (2009) based on standardized major axis regression using “*smart”* package (Warton, Duursma, Falster, & Taskinen, 2012). The effect of the release distance on the probability of returning to the nest was tested using a Cox proportional hazards regression model from “*survival”* package (Therneau, 2014). The significance of the overall model including the standardized mass of the hornets and the cardinal point of release was tested using log‐likelihood ratio test. As expected in homing experiments, some individuals (*n* = 205) were still missing at the end of the experiment (minimum time since their release = 320 hr) and were thus included as censored data. A Tukey post hoc test was used to test the differences among groups (distance and/or cardinal points). The difference in body condition between the hornets that returned to the nest and those that did not come back was tested with Wilcoxon rank sum test.

Individual activity was then analyzed based on the individuals, which were released under the nest (it includes the 30 individuals released at the nest in the homing activity test and the 41 supplementary individuals. *N* = 71, Supporting Information Appendix S3) to avoid potential confounding effect of the consequences of flying over long distance. The influence of the weather conditions (temperature, wind, and humidity, obtained by the platform INRA CLIMATIK) on the number of trips per day per individual was assessed using a negative binomial generalized linear mixed effects model (NBGLMM). First, a synthetic variable including the mean daily temperature (mean ± *SD* during the experiment: 26.11 ± 4.35°C), humidity (46.94 ± 13.80%), and wind speed (4.26 ± 1.07 m/s) was computed with principal component analysis (PCA). The first axis of the PCA (PC1) accounting for 60.71% of the total variance (eigenvalue >1) was therefore used to describe the daily weather conditions (factors loadings: temperature: 0.70; humidity = −0.70; wind speed: 0.14); positive values of PC1 correspond to warm dry and windy days, while negative ones correspond to mild and humid days. The NBGLMM included the identity of the individual as random effect. The number of trips per day and hour was also compared among individuals using Poisson generalized linear model (GLM) including quadratic effects for days and hours (see Monceau, Arca, et al., 2013). For GLM and NBGLMM, the statistical significance of each parameter was tested with likelihood ratio‐based *χ* ^2^ statistics and Wald test, respectively, for unbalanced design (Fox & Weisberg, 2011).

The length of each trip was extracted by the automated analysis of RFID tracking data Track‐a‐forager software (v 1.0, Van Geystelen, Benaets, de Graaf, Larmuseau, & Wenseleers, 2016). Different parameters were selected: (a) “natural foraging,” because no food source was installed outside, (b) “shared access” for in and out access, because entering and leaving the nest are performed through the same pathway, and (c) “two” portals*, that is,* the total number of portals installed. Trips shorter than 20 s and longer than 86,400 s (i.e., 24 hr) were not included, and the minimal threshold length was fixed to 60 s. The effect of individual body condition on the trip length was tested using linear mixed effects models (LMMs) based on rank transformation, associated with *F*‐ratio statistics. This procedure was preferred to the classical nonparametric Friedman tests because the data did not meet the conditions of normality and homoscedasticity (Baguley, 2012).

## 3 RESULTS

### 3.1 Homing ability of *V. velutina* workers

**Figure 2:**
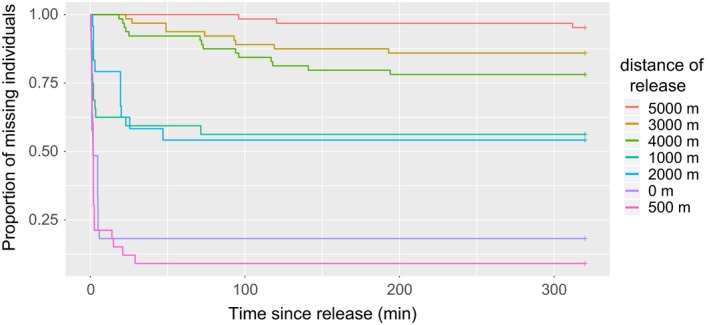
Homing time to nest of tagged workers of *Vespa velutina* in function of their release distance from nest. *N* = 318

No difference between the two replicates of the 3,000, 4,000, and 5,000 m release distances in the number of individuals coming back to the nest was detected (Fisher's exact test for 3,000 m: *p* = 1 and 5,000 m *p* = 0.24; Pearson's *χ* ^2^ for 4,000 m: *χ* ^2^ = 0, *df* = 1, *p* = 1). Thus, the two sessions for each distance were pooled for subsequent analyses. All distances pooled, and a total of 112 individuals over 318 released individuals were detected back at the nest (Table 1, Figure 2). However, four of them (released near the nest, i.e., 0 m) were excluded from the following analyses because their return was not recorded (only the first exit after the return). The probability of returning to the nest was affected by the distance of release (Cox proportional hazard model: *χ* ^2^ = 161.69, *df* = 6, *p* < 0.0001, Figure 2) but not the body condition of the hornets (*χ* ^2^ = 2.82, *df* = 1, *p* = 0.09) or by the orientation (cardinal points) of the release (*χ* ^2^ = 2.97, *df* = 3, *p* = 0.39). Three different groups based on the release distances did not differ: 0 and 500 m (Tukey's test, *p* = 0.95); 1,000 and 2,000 m (*p* = 1); and 3,000, 4,000 and 5,000 m (*p* > 0.15 in all cases). These groups differed from each other (*p* < 0.05) except in the case of 1,000 versus 4,000 m that is marginally nonsignificant (*p* = 0.08). The homing rate decreased of ca. 50% from a group distance to the further one (Figure 2). Hornets coming back to the nest and those considered lost (i.e., that did not return to the nest during the experiment) differed in their body condition (Wilcoxon rank sum test: *W* = 12,934.5, *p* = 0.01): the returning hornets were lighter (median [95%CI]: 284.1 [273.3; 294.1] mg) than the not returning hornets (295.8 [286.8; 299.9] mg).

**Table 1:** Homing rate, time to return, and speed of *Vespa velutina* workers in function of their release distances, and the cardinal points of release (NE = northeast, NW = northwest, SE = southeast, SW = southwest). The sample size for each category is also given (*N*). *SD*, standard deviation. Homing speed is calculated as time needed by worker to return to the nest. Flying speed in *V. velutina* is unknown, but for comparison in *V. crabro,* flying speed in straight line has been estimated at 1.86 m/s (i.e., 6.7 km/hr) (Spiewok & Schmolz, 2005)

Release distance (m)	*N*	Homing rate (%)					Homing time (hr) Mean ± *SD*	Homing speed (m/hr) Mean ± *SD*
		NE	NW	SE	SW	Overall		
0	71	—	—	—	—	83.78	2.40 ± 2.01	—
500	32	100.00	75.00	12.5	100.00	90.91	3.91 ± 6.73	484.8 ± 596.64
1,000	32	37.50	50.00	25.00	62.50	43.75	8.02 ± 19.17	862.17 ± 691.25
2,000	32	62.50	37.5	62.50	37.50	50.00	16.75 ± 12.21	375.27 ± 451.97
3,000	64	12.50	12.5	18.80	12.50	14.06	80.11 ± 53.23	56.60 ± 39.68
4,000	64	18.80	25.00	25.00	18.80	21.88	77.53 ± 53.34	92.17 ± 73.03
5,000	64	6.25	6.25	0.00	6.25	4.69	176.17 ± 118.3	36.53 ± 18.53

### 3.2 Individual flight activity

Considering the 71 individuals released at the nest (4,467 trips), the individual average duration period of activity lasted 4.98 ± 4.44 days (mean ± *SD*, range: 1–26 days of detection) with an average 12.62 ± 10.97 trips per day per individual. Ninety‐eight percent of the trips were recorded between 7:00 a.m. and 08:00 p.m.; the remaining trips (72) made during the night were excluded from subsequent analyses.

**Figure 3:**
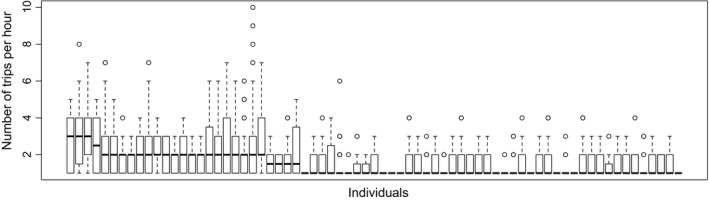
Number of trips per hour of the different *Vespa velutina* workers. Boxes, plain line, dashed lines, and open circles represent 50% of all values, medians, 1.5 interquartile range, and extreme values, respectively

**Figure 4:**
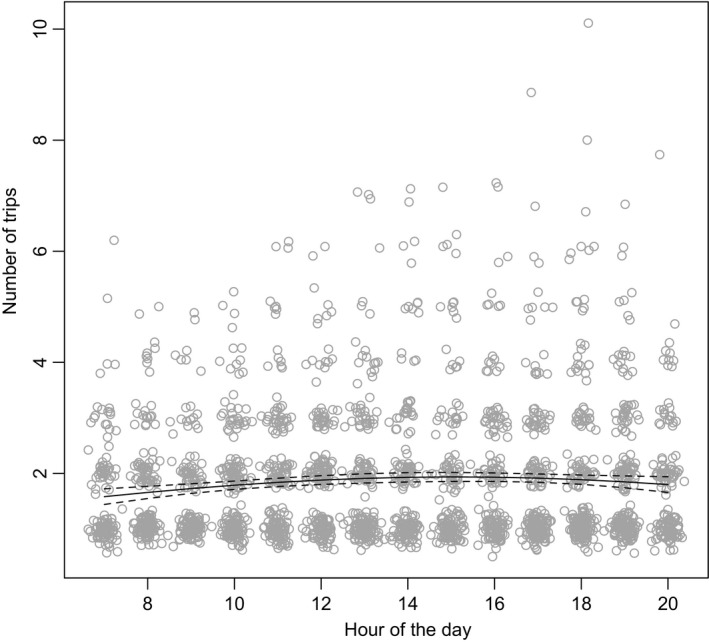
Number of trips of the different *Vespa velutina* workers in function of the hour in the day. Predicted values fitted with the GLM model (plain line) with 95% confidence interval (dash lines)

The number of trips per day and individual were affected by the weather conditions (NBGLMM, estimates ± *SD* = 0.15 ± 0.04; Wald test: *χ* ^2^ = 13.15, *df *= 1, *p* < 0.001): The number of trips increased with higher temperature and lesser humidity. The number of trips differed among individuals with no clear pattern (Poisson GLM: *χ* ^2^ = 353.85, *df* = 70, *p* < 0.0001, Figure 3), and hours of the day (hours: *χ* ^2^ = 17.61, *df* = 1, *p* < 0.0001; hours^2^ *χ* ^2^ = 15.94, *df* = 1, *p* < 0.0001) with a maximal number of trips was reached in early afternoon (02:00 p.m.–03:00 p.m., Figure 4). There was no difference among days (days: *χ* ^2^ = 2.53, *df* = 1, *p* = 0.11; days^2^: *χ* ^2^ = 0.55, *df* = 1, *p* = 0.46) or their interactions (hours × individuals: *χ* ^2^ = 40.73, *df* = 61, *p* = 0.98; hours^2^ × individuals: *χ* ^2^ = 39.55, *df* = 61, *p* = 0.98; days × individuals: *χ* ^2^ = 41.96, *df* = 48, *p* = 0.72; days^2^ × individuals: *χ* ^2^ = 46.61, *df* = 48, *p* = 0.53).

**Figure 5:**
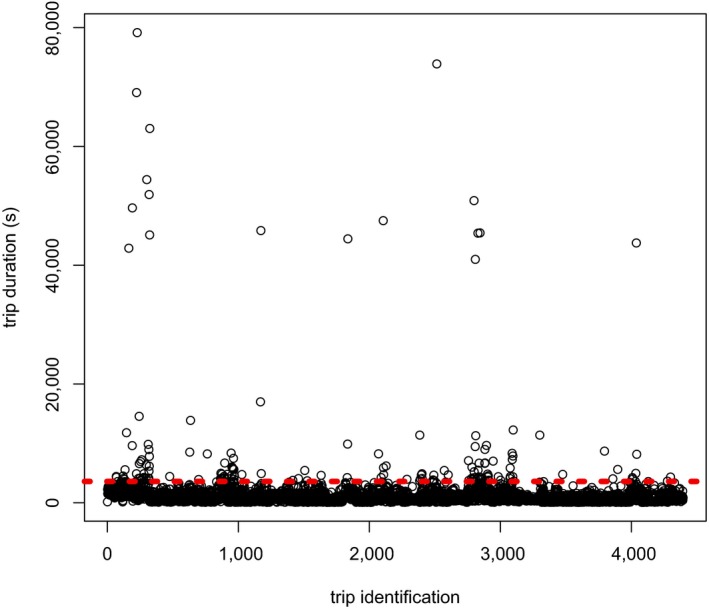
Global trip duration in *Vespa velutina* workers. The red dotted line at 3,600 s = 1 hr, separates short trips from long trips in our analysis

Trip duration was divided into two samples: long trips that lasted more than 1 hr and short trips that lasted less than 1 hr (Figure 5). Long trips represented 3.60% of the trips and range more than 1 hr to ca. 22 hr. These trips were not considered in the following analyses. Most of the trips were thus short trips of ca. 949.7 ± 750.46 s (mean ± *SD*, ca. 15 min 50 ± 12 min 30, range: 68 s to 3,597 s). Trip duration was not influenced by body mass (*F* = 0.34, *df* = 1 and 24, *p* = 0.57).

### 3.3 Anecdotic but noticed behaviors

Twenty‐nine individuals (40% of the 71 tagged individuals used for this analysis) did at least one long trip, lasting more than 1 hr : 24 of them only made one to four long trips in total, two of them made between five and 14 such trips, and three of them did more than 15. Those last three individuals made, respectively, 38, 33, and 23 long trips; 84% of these long trips lasted between 60 and 250 min, with an average of 99 ± 75 min, and 16% of the long trips lasted average 886 ± 193 min.

## 4 DISCUSSION

Daily rhythm of activity and duration of foraging paths are critical behaviors for central place forager (Bell, 1990; Orians & Pearson, 1979). In the present case, the invasive *V. velutina* expand very quickly to different European countries, in which it exerts a very strong predation on honeybees, wild bee, and wasp colonies (Tan et al., 2007; Monceau et al., 2014). Developing monitoring tools like the RFID is thus urgent and of primary importance to evaluate the magnitude of the predation niche around the predator colony. Even though behavioral differences might be expected from individuals from different colonies, the present study was based on the observation of a maximum of individuals, from a single colony considered as a study population which was trained to a foraging niche. This allows having an important batch of tagged insects providing reliable rhythm of activity.

In order to avoid any learning during the outward trip, workers were transported blind to different locations (distance and cardinal points) to evaluate their return capacities. In such conditions, most of the individuals flew back when released up to 500 m, half of them returned to the nest when released up to 2,000 m, and less than a quarter were retrieved when released farther than 3,000 m. These data suggest that *V. velutina* workers can find their way back over several kilometers. Even if it was only evaluated on one colony, it could give us an estimation of this parameter, which could vary in function of colony needs, population size, landscape structure, and resource availability. However, their foraging range is probably lower than 2,000 m, probably in a radius around the nest of 500 m. These results are congruent with the data available in other *Vespa* sp. Indeed, homing ability in *V. orientalis* is ca. 1,000 m with a probable territory range of 500 m (Ugolini, Kessler, & Ishay, 1987). *V. orientalis* and *V. velutina* being of similar size, finding congruent capacity is not surprising. In the case of *V. velutina*, almost 5% of the individual released at 5,000 m were able to find their nest. This long distance can be compared to the foraging range of the giant Japanese hornet *V. mandarinia*, which is ca. 1,000–2,000 m, with a maximal distance of 8,000 m (Matsuura & Sakagami, 1973). This experiment also shows that homing behavior is not affected by the orientation of the release point. This suggests that compass information due to sun orientation or magnetic fields might not be involved in this homing behavior, what is congruent with previous findings in Vespidae. Orientation system mostly relies on visual (Zeil, 1993), olfactory cues (Takagi, Hirose, & Yamasaki, 1980). Vespids learn visual information during an orientation flight, to find their path to or back from foraging sites (Ugolini, 1987; Raveret Richter & Jeanne, 1991; see Raveret Richter, 2000 for a review). In this orientation flight, the individual flies along ever‐increasing arcs around the nest that allow combining flight trajectory (arcs) and gaze orientation to acquire sufficient visual cues for homing (Stürzl et al., 2016; Toh & Okamura, 2003; Zeil, 1993). One should, however, consider that workers’ previous experience was not controlled in our experimental design so some individuals might have already experienced long trips from their nest that could have facilitated their return. Returning workers had a smaller body index (i.e., lower mass for a similar size) that could reflect their age. Indeed, worker body mass increases through the season probably because the consecutive cohorts of workers benefit from increasing food provided during larval stage (Matsuura & Yamane, 1990; Monceau, Bonnard, et al., 2013). Individual with lower body mass could be older individuals, thus with more experience. Most of the tagged workers come back to the nest within the first 24 hr. However, some of them return to the nest more than 4 days (i.e., 100 hr) after their release. Such duration was also observed in *Bombus terrestris* (Goulson & Stout, 2001). This means that individuals may survive for several days outside their nest and thus questions their travel path during this episode. Considering that *V. velutina* nest density in Bordeaux suburbs is quite high, one hypothesis is that individuals may have wandered from colonies to colonies as several non‐nest mates were observed as accepted by other colonies (K. Monceau and O. Bonnard, pers. obs.). It also questions the fate of those that never come back to the nest: lost, died, or fully accepted by other colonies. However, this question cannot be answer with the RFID technique and other tracking devices are still not usable to cover such long distances (see Milanesio, Saccani, Maggiora, Laurino, & Porporato, 2016, 2017).

Up to date, only direct observations or video records have allowed monitoring the activity of *V. velutina* (Monceau, Arca, et al., 2013 2017; Monceau, Bonnard, et al., 2013; Monceau et al., 2017; Perrard, Haxaire, Rortais, & Villemant, 2009). Our results are in line with these previous studies. First, most of the activity is realized between 07:00 a.m. and 08:00 p.m., confirming that *V. velutina* is diurnal; some individuals still have a nocturnal activity (only 2% of the activity). *V. crabro* is also active with low light intensity but in a higher propensity (Kelber et al., 2011). Second, the worker activity is driven by weather conditions that is quite classical in Vespidae (Canevazzi & Noll, 2011; de Castro, Guimaraes, & Prezoto, 2011; Cruz, Giannotti, Santos, Bichara Filho, & Resende, 2006; Kasper, Reeson, Mackay, & Austin, 2008; da Rocha & Giannotti, 2007). The observed enhancement of the hornet activity during the day with a maximum around noon, already observed by video analysis (Monceau et al., 2017), can be either attributed to an increase in temperature or in UVB solar irradiation. Indeed, *V. orientalis* is able to convert solar into metabolic energy with photovoltaic like cuticle cells (Ishay, 2004; Ishay & Kirshboim, 2000; Plotkin et al., 2010; Volynchik, Plotkin, Bergman, & Ishay, 2008). Such a reaction has not been investigated in *V. velutina* for now but should receive attention as it would also explain its performance in hovering for preying honeybees (Monceau, Arca, et al., 2013).

Contrary to previous studies, RFID allows identifying unique individual behavior. Thus, the duration of each trip can be accurately quantified with RFID: 95% of the flights lasted less than 1 hr. Flying speeds of *V. velutina* workers are so far unknown, but in *V. crabro*, it has been estimated at 1.86 m/s (i.e., 6.7 km/hr) (Spiewok & Schmolz, 2005). If both species fly at a similar speed and considering the average trip duration being 15 min, *V. velutina* workers probably forage within less than 1,000 m away from their nest. Moreover, predation includes catching and processing the prey and then coming back to the nest with an additional load that impacts the flying speed; thus, they probably forage in a 500–800 m diameter perimeter. This means that if predation is detected on hives, *V. velutina* nest should be searched within a radius of at least 1,000 m. However, this approximation is based on a specific area where resources (i.e., honeybee hives) are common and thus should be replicated in a different area. Nevertheless, it is congruent with the homing behavior.

Few hornet foragers realized in this study particularly long trips. These individuals could be considered as elite foragers or scout individuals such as in bees characterized by a strong explorative capacity (Degen et al., 2015; Grüter, Leadbeater, & Ratnieks, 2010). This was already described in vespids (Roberson, Nordheim, & Jeanne, 2003): They observed a bimodal repartition of the workers of *Vespula germanica*, few of them making disproportionate number of trips. In bees, the proportion of such atypical foraging behaviors in a colony varies with colony condition and also environmental stressors (Grüter et al., 2010; Klein, Cabriol, Devaud, Barron, & Lihoreau, 2017). Such observations should be confirmed in *V. velutina* but may be important in trying understanding the capacity of workers to explore novel terrain around their colony and thus the niche size of a colony.

This work explored for the first time homing abilities using RFID technic in an invasive hornet species threatening honeybees, and allowed us to evaluate the boundaries of its foraging range. Harmonic radar allows tracking hornets only over short distances, but is not suited to follow several signals at the same time, and it is not accurate enough (Milanesio et al., 2016, 2017): The RFID provides the best compromise to acquire new information on workers’ flight behavior that are of first interest for the monitoring and control of this special invasive hornet. Invasive social insects, especially vespids, can deeply affect their environments (Beggs et al., 2011; Bradshaw et al., 2016), and their impact is obviously related to foraging range. How animals use their environment and their movements is key parameters in biological invasion (Holway & Suarez, 1999), and such parameters should be implanted in future impact models. The development of the RFID techniques to study hornets will provide a very useful tool for comparing activity ranges of workers in different ecological conditions. For example, this would allow determining the impact of treatments or parasitism by entomopathogens on homing capacities, action range, and activity rhythm in *V. velutina* workers (Poidatz, Javier Lopez Plantey & Thiéry, 2018). This technique would also be very helpful in understanding the role of pesticide accumulation on the hornets foraging activity and in managing the hive protection in areas colonized with *V. velutina*.

## CONFLICT OF INTEREST

The authors declare no conflict of interest.

## AUTHOR CONTRIBUTIONS

JP, OB, and DT conceived the ideas and designed methodology; JP and OB collected the data; JP and KM analyzed the data; and JP, DT, and KM wrote the manuscript.
