# Supplementary material for: Activity rhythm and action range of workers of the invasive hornet predator of honeybees Vespa velutina, measured by radio frequency identification tags
Source: Ecol Evol. 2018 Jul 8;8(15):7588–98. doi: 10.1002/ece3.4182 (PMC6106190; doi:10.1002/ece3.4182)
Supplement: Supplementary file 1 [file ECE3-8-7588-s001.docx]

**Appendice S1. Installation of the nest within the cage inside the cabin (side view).** The nest is fixed with iron strings inside the cage made of mahogany stainless steel grid and Plexiglas. This cage was equipped with two secured apertures and one secured covered sliding plate and a mobile drawer for food and water supply. The cage is place on a table inside a 2m x 1.5m x 2m stainless steel grid cabin with a corrugated plastic roof.

Once allowed to leave the cage, the hornets can access the outside or enter the nest *via* a 10 cm diameter transparent plastic tunnel connecting the cage to the outside. The RFID portals, covered with a plastic copper (see details in Appendice S2), are positioned at one extremity of the tunnel so the hornet must pass through the portals to go in and out. All movements are recorded by A RFID host controller iID® HOST MAJA (Mycrosensys).

**
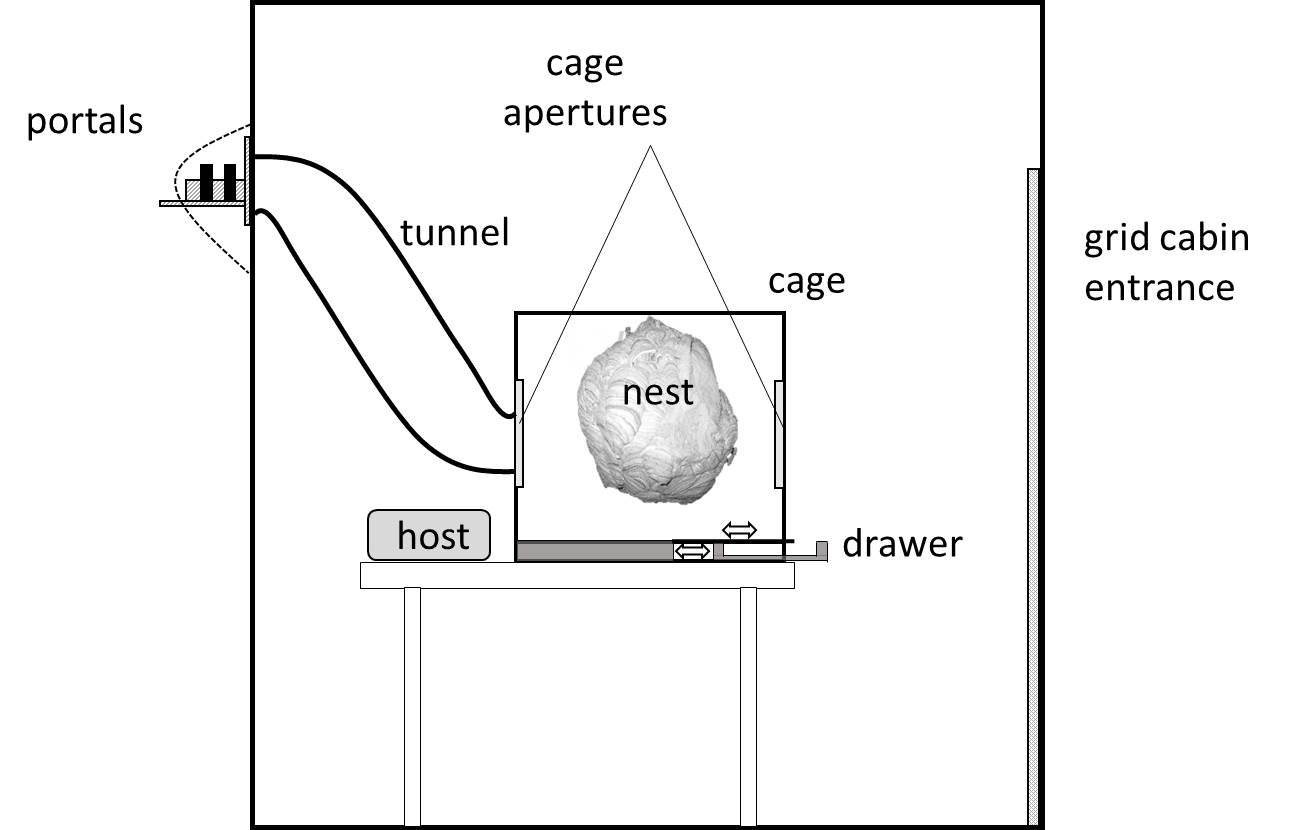
**

**Appendice S2. RFID portal design.** Two RFID portals A and B (MAJA® reader module 4.2, Mycrosensys) were placed on a 5 cm long wood support, one behind another at the entrance of the tunnel on the outside. Thus ongoing (AB sequence) and outgoing individuals (BA sequence) could be sorted out and are recorded by the RFID host controller (Appendice S2). Hornets are tagged with RFID micro TAG (mic3®-TAG 16Kbit, iID-2000-G, 2.0x1.7x0.5mm). We tested the percentage of misreading of Tags by the portals at every release session, by using tags fixed on a stick and pass it through the portals (N=30). We observed a reading efficiency of 95%.

**From the side**


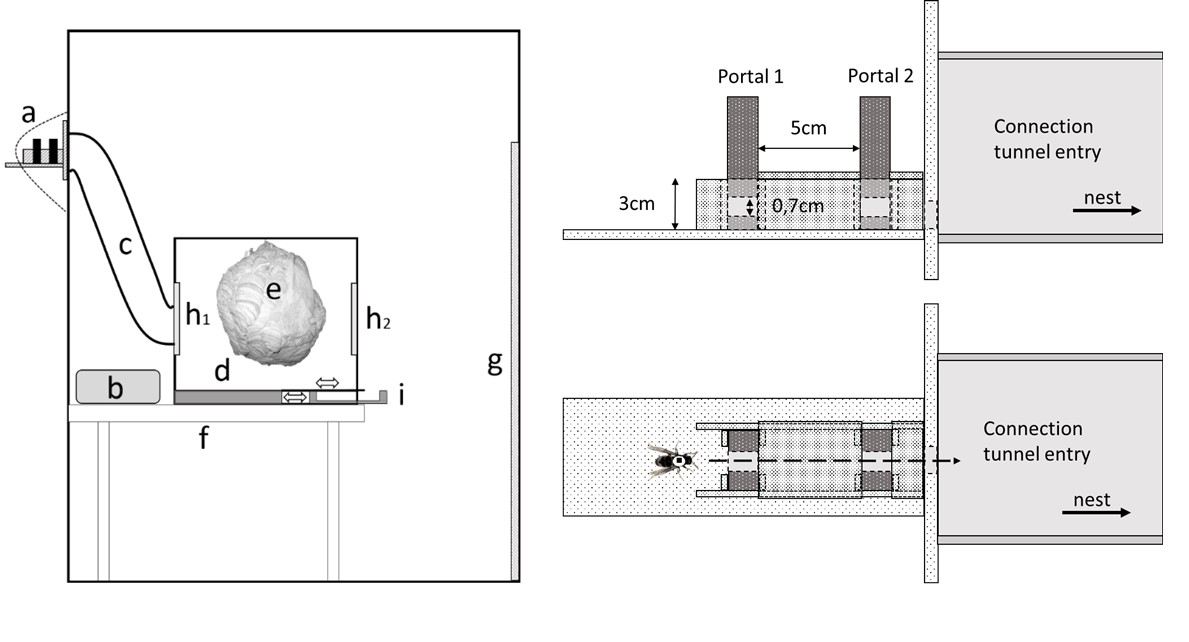


**From above**


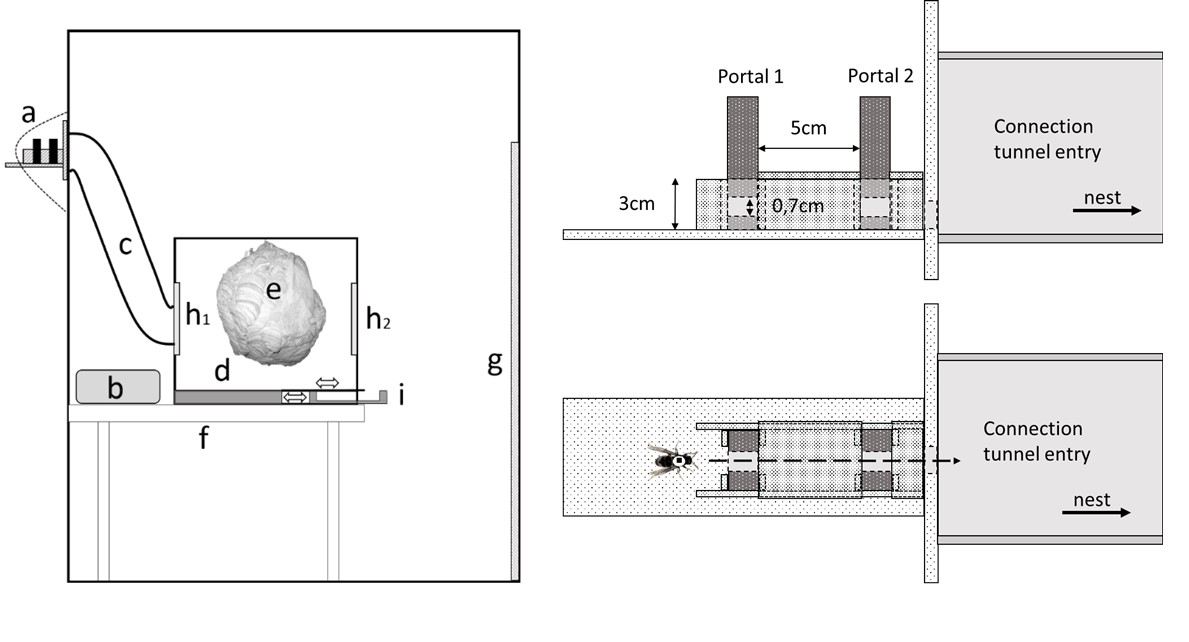


**Appendice S3. GPS coordinates of the different release points of the hornets at different distances from the nest.** For 3000, 4000 and 5000m distances of release, 2 replicates were made.

| Distance (m) | Cardinal point | GPS coordinates | Date | Number of hornets |
| --- | --- | --- | --- | --- |
| 0 | - | 44°47'30.4"N 0°34'36.9"W | 5 August 2016 | 5 |
|  |  |  | 8-10 August 2016 | 36 |
|  |  |  | 30 August 2016 | 30 |
| 500 | NW | 44°47'37.6"N 0°34'32.3"W | 16 August 2016 | 8 |
|  | SE | 44°47'16.2"N 0°34'24.8"W | 16 August 2016 | 8 |
|  | SW | 44°47'08.0"N 0°34'35.8"W | 16 August 2016 | 8 |
|  | NE | 44°47'33.2"N 0°34'55.6"W | 16 August 2016 | 8 |
| 1000 | NE | 44°47'20.4"N 0°33'49.1"W | 16 August 2016 | 8 |
|  | SE | 44°46'55.5"N 0°34'16.2"W | 17 August 2016 | 8 |
|  | NW | 44°47'13.1"N 0°35'30.8"W | 17 August 2016 | 8 |
|  | SW | 44°47'49.3"N 0°35'13.2"W | 17 August 2016 | 8 |
| 2000 | NE | 44°47'36.1"N 0°33'12.4"W | 28 September 2016 | 8 |
|  | SE | 44°46'42.4"N 0°35'12.1"W | 28 September 2016 | 8 |
|  | SW | 44°47'31.3"N 0°36'02.0"W | 28 September 2016 | 8 |
|  | NW | 44°48'38.7"N 0°35'04.7"W | 28 September 2016 | 8 |
| 3000 | NE | 44°48'08.8"N 0°32'34.9"W | 18 August 2016 | 8 |
|  |  |  | 10 October 2016 | 8 |
|  | SE | 44°45'50.4"N 0°34'03.7"W | 18 August 2016 | 8 |
|  |  |  | 10 October 2016 | 8 |
|  | SW | 44°46'18.2"N 0°36'22.1"W | 18 August 2016 | 8 |
|  |  |  | 10 October 2016 | 8 |
|  | NW | 44°48'47.4"N 0°34'58.3"W | 18 August 2016 | 8 |
|  |  |  | 10 October 2016 | 8 |
| 4000 | NE | 44°47'43.9"N 0°31'41.0"W | 29 September 2016 | 8 |
|  |  |  | 11 October 2016 | 8 |
|  | SE | 44°45'38.1"N 0°33'03.7"W | 29 September 2016 | 8 |
|  |  |  | 10 October 2016 | 8 |
|  | SW | 44°45'33.2"N 0°35'57.3"W | 29 September 2016 | 8 |
|  |  |  | 10 October 2016 | 8 |
|  | NW | 44°48'53.0"N 0°36'32.8"W | 29 September 2016 | 8 |
|  |  |  | 11 October 2016 | 8 |
| 5000 | NE | 44°49'08.8"N 0°32'20.5"W | 18 August 2016 | 8 |
|  |  |  | 10 October 2016 | 8 |
|  | SE | 44°44'51.7"N 0°33'53.2"W | 18 August 2016 | 8 |
|  |  |  | 10 October 2016 | 8 |
|  | SW | 44°45'14.8"N 0°37'31.5"W | 18 August 2016 | 8 |
|  |  |  | 18 October 2016 | 8 |
|  | NW | 44°48'39.9"N 0°37'42.7"W | 18 August 2016 | 8 |
|  |  |  | 18 October 2016 | 8 |
